# TrajectoryGeometry suggests cell fate decisions can involve branches rather than bifurcations

**DOI:** 10.1093/nargab/lqae139

**Published:** 2024-10-08

**Authors:** Anna Laddach, Vassilis Pachnis, Michael Shapiro

**Affiliations:** Nervous System Development and Homeostasis Laboratory, The Francis Crick Institute, 1 Midland Rd, London NW1 1AT, UK; Nervous System Development and Homeostasis Laboratory, The Francis Crick Institute, 1 Midland Rd, London NW1 1AT, UK; Nervous System Development and Homeostasis Laboratory, The Francis Crick Institute, 1 Midland Rd, London NW1 1AT, UK

## Abstract

Differentiation of multipotential progenitor cells is a key process in the development of any multi-cellular organism and often continues throughout its life. It is often assumed that a bi-potential progenitor develops along a (relatively) straight trajectory until it reaches a decision point where the trajectory bifurcates. At this point one of two directions is chosen, each direction representing the unfolding of a new transcriptional programme. However, we have lacked quantitative means for testing this model. Accordingly, we have developed the R package TrajectoryGeometry. Applying this to published data we find several examples where, rather than bifurcate, developmental pathways *branch*. That is, the bipotential progenitor develops along a relatively straight trajectory leading to one of its potential fates. A second relatively straight trajectory branches off from this towards the other potential fate. In this sense only cells that branch off to follow the second trajectory make a ‘decision’. Our methods give precise descriptions of the genes and cellular pathways involved in these trajectories. We speculate that branching may be the more common behaviour and may have advantages from a control-theoretic viewpoint.

## Introduction

Multicellular organisms consist of complex communities of diverse cell types. Remarkably, these all arise from a single cell, the zygote. Accordingly, the development of an organism involves cell fate restriction and differentiation. It has been shown that cell differentiation frequently proceeds via successive binary decisions (1), although multifurcations are also possible (2). Here, we will focus on binary cell fate decisions. A typical model of these cell fate decisions is as follows: a multi-potential progenitor cell develops until it reaches a decision point. Here, the cell chooses one of two possible fates, each of which requires the initiation of a new developmental programme. After making this choice the cell develops towards the chosen fate. We refer to this as the *bifurcation model*, where each chosen outcome is seen as more differentiated than the progenitor state.

**Figure 1.**
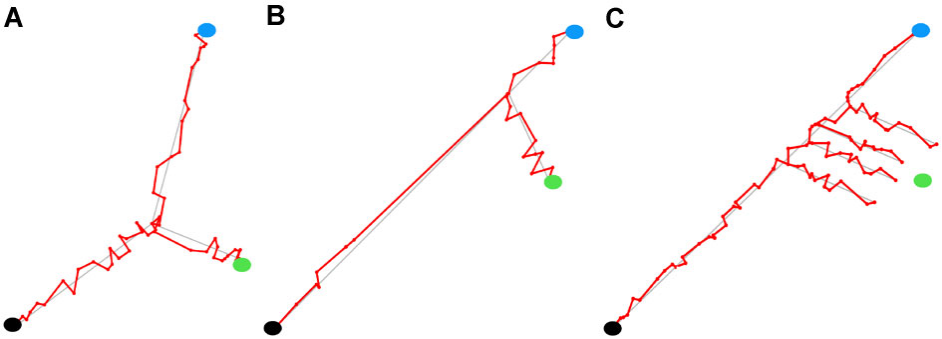
Synthetic data showing bifurcation and branching models. (**A**) Bifurcation as a model of cell-fate decision. Bi-potential progenitor cells (black) proceed to a decision point after which they proceed in one of two new directions in gene expression space. (**B**) A simplified version of branching behaviour as a model of cell-fate decision. Here bi-potential cells proceed along a default developmental pathway to one of their potential outcomes. The decision is whether or not to leave this default pathway and develop in a new direction. (**C**) In this version of branching behaviour, there is a region rather than a single point at which cells choose to leave the default pathway.

Our exploration of lineage decisions in the enteric nervous system (ENS)(3) has uncovered a novel configuration of differentiation trajectories. In contrast to the bifurcation model, enteric gliogenesis forms a default ‘linear’ path of progenitor maturation, from which neurogenic trajectories branch off during embryogenesis. A consequence of this branching configuration is that there are no identifiable points of commitment along the gliogenic trajectory and it is only the cells which become neurons that ever ‘make a decision’ and initiate a new developmental program. Further, rather than a single branch point, there seems to be a region along the default trajectory where this branching can take place. These models are portrayed in Figure 1. We have suggested this branching model of lineage decisions allows for plasticity along the default trajectory and underpins the neurogenic potential of mature enteric glial cells.

Our analytic tool for observing default and branching behaviour is our Bioconductor package TrajectoryGeometry(4). The asynchronicity of most differentiation processes enables the simultaneous profiling of cells at different positions along their developmental trajectory. Many packages exist to infer pseudotime trajectories (5–7), and all of these consider the geometry of the cells and their clusters in gene expression space. There are also packages to discover the genes that are differentially expressed over pseudotime (8). However, to the best of our knowledge, TrajectoryGeometry is the first to analyse the pseudotime trajectories and gene expression changes along these trajectories using their overall geometry. The notion of a default trajectory is not new, (see (9) and below). However, TrajectoryGeometry provides an analytic footing for this idea by detecting whether a developmental trajectory proceeds in a well-defined direction.

Having observed a branching model of lineage decisions in the development of enteric neurons and glial cells, we were led to question whether this behaviour is unique to the ENS or whether it might be employed more generally. Here we show that this behaviour is observed in the development of hepatocytes into hepatoblasts and cholangiocytes (9) and the development of postnatal murine olfactory stem cells into into sustentacular cells, neurons and microvillous cells (10) (Supporting Information).

## Materials and methods

Trajectory geometry can infer bifurcating and branching models of cell fate decisions by testing whether a trajectory proceeds in a well defined direction. If bifurcation is in operation both trajectories change direction after the decision point (Figure 1A), whereas in the branching case only one trajectory changes directionality at this point (Figure 1B); moreover there may be a region along the default trajectory from which branching can take place (Figure 1C).

A direction in two dimensions is a point on a circle, e.g., a compass point. A direction in three dimensions is a point on the sphere. More generally, a direction in *N* dimensions is a point on the *N* − 1 dimensional sphere, $\mathbb {S}^{N-1}$. A path with a well-defined directionality gives rise to points on the circle, the sphere, or $\mathbb {S}^{N-1}$ which are tightly clustered around their common center (Supplementary Figure S1). This allows TrajectoryGeometry to detect whether a differentiation trajectory has a well-defined directionality, produce a *P*-value for that directionality and specify its direction. This direction identifies the genes that are up- and down-regulated along the trajectory and consequently which biological pathways are being up- and down-regulated. Further details are given in (3), in the documentation accompanying (4) and in the supporting information.

## Results

### Branching cell fate decisions in the liver

**Figure 2.**
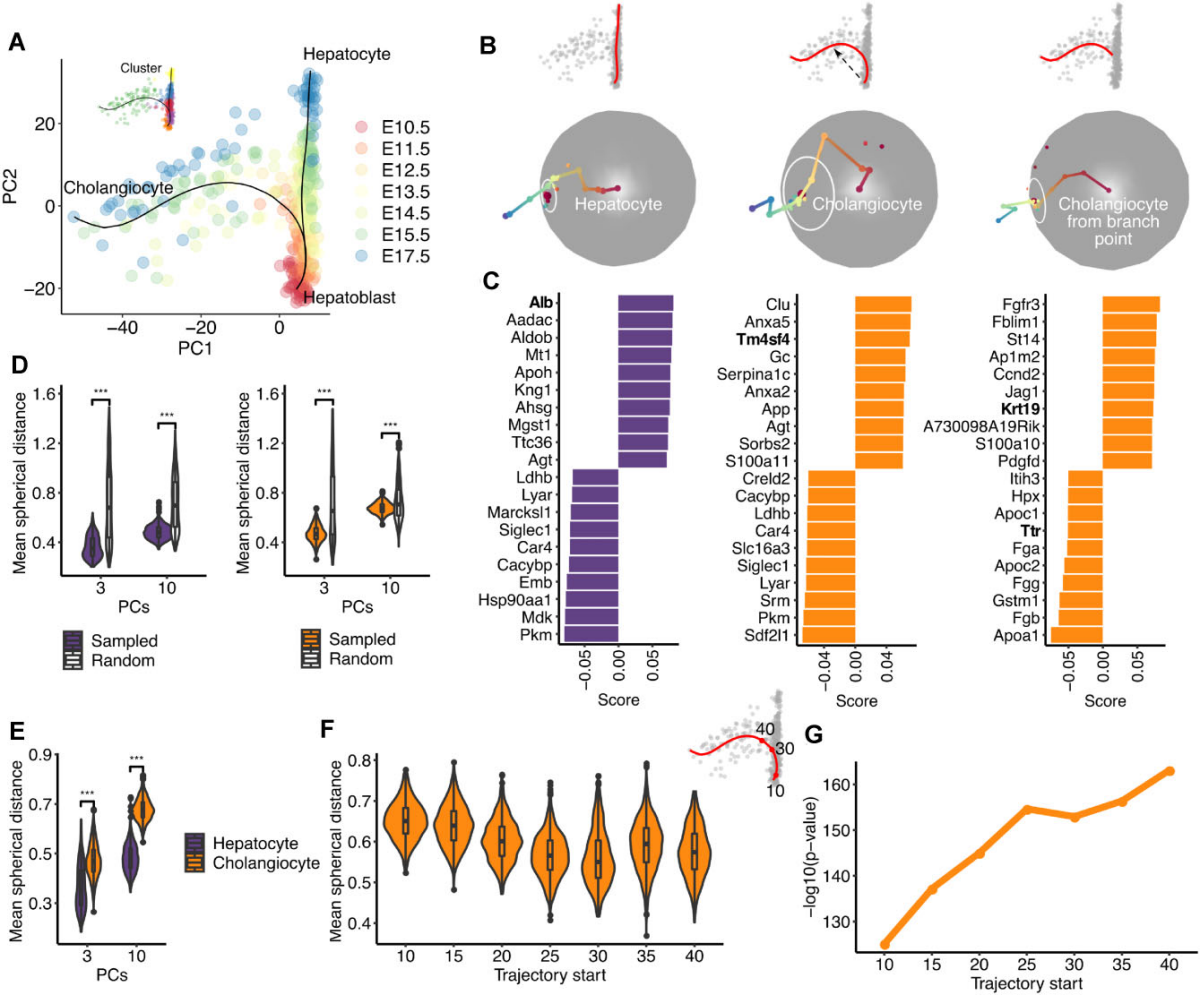
(**A**) PCA plot of scRNAseq data for embryonic murine hepatobiliary cells. Pseudotime trajectories inferred using Slingshot are shown on the plot. Cells are coloured by Louvain cluster. (**B**) 3D sampled pathways for hepatoblast to hepatocyte, hepatoblast to cholangiocyte, and decision point to cholangiocyte trajectories together with their projections on the 2-sphere. White circles denote mean distance from center (red dot). (**C**) Bar plots showing top 10 up- and down-regulated genes for each trajectory as in (B), using 10 dimensions. (**D**) Violin plots indicating the mean spherical distance (radii of the white circles in B) for paths sampled from the hepatocyte and cholangiocyte trajectories (purple and orange, respectively) relative to random trajectories (white). Statistics calculated using 1000 random paths from each trajectory and the first 3 and the first 10 PCs respectively and the first 3 and the first 10 PCs respectively. (**E**) Violin plots indicating the mean spherical distance of the hepatocyte (purple) and cholangiocyte (orange) trajectories. (**F**) Violin plots indicating the mean spherical distance for the cholangiocyte trajectory (first 3 PCs) starting from successively later points in pseudotime, as the decision point is approached (30 value on the cholangiocyte trajectory shown in the top right inset). (**G**) Line graph indicating the –log_10_(*P*-value) for the significance of directionality for the cholangiocyte trajectory (first 3 PCs) relative to random trajectories, starting from successively later points in pseudotime.

In the liver, hepatoblasts give rise to hepatocytes and cholangiocytes (Figure 2A). Here, we analyse murine single cell data describing this process from (9). Branching is clearly visible in the 2D visualisation of pseudotime trajectories, as noted in (9), Figure 2A, where the trajectory that gives rise to cholangiocytes appears to branch off a default time-axis aligned trajectory that ultimately generates hepatocytes. Using TrajectoryGeometry, we see that the small circle observed for the hepatocyte trajectory, in comparison to the larger circle observed for the cholangiocyte trajectory (Figure 2B), suggests hepatocyte development maintains a relatively consistent directionality of gene expression change. Conversely, if the cholangiocyte trajectory is analysed from the decision point onwards, a small circle is also observed, suggesting that it maintains a consistent directionality after branching off (Figure 2B).

Sampling 1000 paths from each trajectory reveals significant directionality in comparison to randomised trajectories for both the cholangiocyte and hepatocyte trajectories (Figure 2D). This is observed whether one uses the first 3 PCs or the first 10 PCs as well as an analysis in full gene expression space (see Supporting Information and Supplementary Figure S2). However direct comparison of cholangiocyte and hepatocyte trajectories reveals that the hepatocyte trajectory maintains a more consistent directionality of gene expression change (Figure 2E)). Furthermore, if the cholangiocyte trajectory segments are analysed starting from successively later points in pseudotime, the mean spherical distance decreases as the decision point is approached and the directionality of the analysed segments becomes more significant (Figure 2F, G), supporting branching behaviour.

Genes positively associated with the directionality of the hepatocyte trajectory include mature hepatocyte markers (e.g. Alb (11)) whereas those negatively associated include markers of hepatoblasts (e.g. Mdk (12)) (Figure 2E). Genes associated with the overall directionality of the cholangiocyte trajectory include those with expression in immature cholangiocytes (e.g. Tm4sf4 (13,14)) (Figure 2c). This appears to result from the overall directionality being a combination of distinct directionalities before and after the decision point (DP). It is only when we look at the trajectory from the DP to the cholangiocytes that we see markers of mature cholangiocytes (e.g. Krt19 (9)) indicating that a directionality that leads to a cholangiocyte phenotype is not achieved until after branching off (Figure 2C). Mature hepatocyte markers (e.g. Ttr (15)) are amongst genes negatively associated with the DP-cholangiocyte trajectory segment (Figure 2C). This indicates that progenitors have already progressed towards a hepatocyte phenotype when they reach the branch point, and these genes must be subsequently downregulated to acquire a cholangiocyte fate.

Interestingly the gene Sox9, which has been reported to be associated with maintaining cholangiocyte fate (16), has a positive score for the DP-cholangiocyte trajectory segment (0.038), but a small negative score for the hepatoblast-DP segment (-0.0045), indicating that it is not associated with the directionality of the trajectory until after the decision point. In contrast, the gene Tgfbr2, reported to be involved in the initial induction of cholangiocyte fate (16), is not amongst the top 2000 highly variable genes used for this analysis, and analysis incorporating all genes (see supporting information) shows only small associations with the directionality of the trajectory (0.0053 hepatoblast-DP, −0.0012 DP-cholangiocyte). Taken together these results show that genes such as Sox9 that are responsible for maintaining cell fate are positively associated with the trajectory from the decision point onwards whereas genes that enable branching, such as Tgfbr2 may show little association with the directionality of the trajectory. We suggest that this is because minimal variation in receptor expression may be required for cells to respond to a fate-inducing signal.

Together these findings further support a model where the cholangiocyte trajectory branches off from a default hepatocyte trajectory. Although the inferred trajectory shows a single branch point, the dispersion of cells around this branch, and the fact that the cells are from different embryonic time points, from E11.5 to E14.5, suggest that branching is possible from a continuous section of the ‘default’ trajectory. The change in the directionality of gene expression at the decision point for the cholangiocyte trajectory signifies initiation of a new transcriptomic programme, suggesting that cells are responding to an extrinsic signal. In agreement with this, the cholangiocyte fate decision has been shown to be coordinately regulated by TGF-beta, WNT, Notch and FGF signalling (17–22) likely in response to factors produced by the periportal mesenchyme.

The liver has remarkable regenerative power, with both cholangiocytes and hepatocytes acting as facultative stem cells able to transdifferentiate if regenerative capacity of the other population is impaired (23). However, it is interesting to note that hepatocytes, that result from the default trajectory, appear to have unlimited regenerative capacity (24). It is also interesting that the most abundant cell type (70% of liver cells are hepatocytes (25)) appears to be produced by default.

### Nested cell fate decisions in the olfactory epithelium

In the Supporting Information, we present an analysis of nested cell fate decisions seen in data from (10), describing differentiation trajectories in the postnatal olfactory epithelium (Supplementary Figures S3–S6). TrajectoryGeometry analysis shows that horizontal basal cells (HBCs) follow a default trajectory to differentiate into sustentacular cells. Neuronal and microvillous trajectories branch off from this trajectory (see Supplementary Figure S3a). A second decision point (DP2) where neuronal and microvillous trajectories diverge appears to be more complex. Initial analysis suggests this is a bifurcation point. However, using TrajectoryGeometry, we discover this is due to the transient overlay of proliferation obscuring branching behaviour. Controlling for this reveals a branch point with neurogenesis as the default trajectory. As microvillous cells are comparatively rare it is parsimonious that these are not produced by default.

### A negative control

In the Supporting Information, we analyse a synthetic data set which exhibits symmetric bifurcation behaviour and show that in this case TrajectoryGeometry does not detect the hallmarks of branching behaviour (Supplementary Figure S7).

## Discussion

In this paper, we have used TrajectoryGeometry to examine the geometry of the cell-fate decisions of multi-potential progenitor cells. Our analyses of several data sets have led us to propose that a branching rather than bifurcating model of cell fate decisions is often employed. In this model, a bipotential progenitor proceeds along a more or less straight *default trajectory* to one of its potential fates. Its other fate arises by *branching off* from this trajectory. In particular, only one of the two cell fates involves initiating a new developmental program. In our experience there is a region along the default trajectory where this branching can take place. Note that development along the default trajectory involves change in gene expression (e.g. the unfolding of a gene expression programme), but not a change in direction of travel in gene expression space as is the case with the initiation of a new programme of gene expression.

Interestingly this model has been anticipated in a more informal manner, e.g. (9) who state: ‘Thus, the default pathway for hepatoblasts is to differentiate into hepatocytes, but along the way, some hepatoblasts are regulated to differentiate toward the cholangiocyte fate.’ TrajectoryGeometry provides the tools to put this in a formal framework by quantifying directionality of trajectories in gene expression space. When it detects directionality, this is expressed as a vector in gene expression space, and this vector tells which genes are most up- and down-regulated in the developmental process. These allow us to detect the functional pathways involved.

So far we have seen three unequivocal cases of branching behaviour: the branching of enteric neurogenic trajectories from the default gliogenic trajectory(3); the branching of cholangiocytes from the default development of hepatoblasts into hepatocytes; and the branching of microvillous and neuronal development at DP1 from the default development of horizontal basal cells into sustentacular cells. The point at which microvillous and neuronal development diverges is slightly more complex. Branching behaviour is obscured by a specific process (the cell cycle), which does not exhibit branching behaviour. However, using TrajectoryGeometry we are able to deconvolute process specific effects, and find that this represents another example of branching behaviour.

Note that there seems to be a defined region along the default trajectory where branching can occur that is permissive for initiation of the new developmental program. The question arises as to whether branching (or the transient upregulation of cell-cycle) arises due to intrinsic or extrinsic signals. Both cholangiocyte development and neuronal branching at DP1 are known to be responsive to WNT signalling. It is possible that only a portion of the default pathway is responsive to external signals. It is also possible that these external signals only arise at specific developmental time points or in specific cellular environments.

Here, we hypothesize that branching behaviour may be more common than bifurcation and have specific evolutionary advantages. Firstly, we speculate that branching is more robust than bifurcation from a control-theoretic viewpoint. When a cell initiates a new transcriptional program there is always an opportunity for error, both in terms of the external signals inducing this change and in terms of the intrinsic pathways induced by these signals. Changing the transcriptional program of only a subset of the cells exposes fewer cells to this danger. This is particularly advantageous when the branching cell type is required in lower numbers, since in this case only a minority of the cells are required to initiate a new transcriptomic program. We have seen that the minority cell type is the branch outcome in the neurons in the ENS, cholangiocytes in the liver and microvillous cells in the olfactory epithelium. Moreover, we hypothesise that branching behaviour allows for simpler coordinate control of cell numbers for two populations, which is particularly desirable when the two cell types function in concert (e.g. glial cells and neurons) and correct proportions must be maintained.

Secondly, branching behaviour may allow for more phenotypic plasticity in the cells along the default trajectory, as this does not involve initiation of a new transcriptomic programme. This appears to be the case in the ENS where mature glial cells retain neurogenic potential which can be activated under certain conditions. Although both cholangiocytes and hepatocytes retain remarkable plasticity in the liver (both populations can transdifferentiate), hepatocytes, the default outcome, maintain unlimited regenerative capacity.

Thirdly, a default trajectory may allow for the faster generation of a differentiated cell type, particularly in cases where cells undergo direct fate conversion and do not reenter the cell cycle. For example, sustentacular cells (generated via direct fate conversion) might be urgently required upon loss to maintain the structural integrity of the olfactory epithelium. Furthermore, it has been suggested that sustentacular cells produce crucial factors for olfactory epithelium regeneration (26); their replenishment might be required before cell types resulting from branching trajectories can be generated.

Although we have suggested several advantages of branching behaviour, one can hypothesise that there are situations in which bifurcation is desirable. For example, in the case where the populations of each of the resulting differentiated cell types must be individually regulated. Here the decision point could consist of a self-renewing population and differentiation into either of the cell fates could be induced by external signals. This contrasts with situations where it is desirable to coordinately regulate the proportions of resulting cell populations.

The Waddington landscape is commonly used as a metaphor to represent cell fate decisions, with undifferentiated cells rolling down valleys to reach basins that represent more differentiated cell fates. Here, a cell rolls down to a decision point where it stochastically chooses one of two valleys. In a branching model of cell fate decisions, we can imagine that the default valley is relatively straight and that it is relatively shallow along the branching region where another valley, with a broad mouth, leads to the alternate outcome

A more sophisticated version of the Waddington landscape is given by the Waddington dynamics of (27,28) where the potential landscape can vary in response to external inter-cellular signals. Note that the landscape exists on a *per cell* basis since individual cells may receive different signals or signals of differing intensities and, indeed, could vary over time scales comparable to differentiation.

Their binary flip landscape can accommodate the two main features of our branching model; the existence of a default and an alternate trajectory and the extended branching region. The first is the geometry of the escape path from the progenitor basin to the default outcome. We hypothesize that under the default signalling, there is a shallow region of the default valley and that the alternate escape route branches off here under the influence of the alternate signal. We suggest that the exact location where this happens depends on the intensity of the alternate signal thus producing a branching region rather than a single decision point.

In the case of enteric neural progenitors, neurons and glia (the default outcome), data suggests there is only a shallow drop from progenitors to glia, as witnessed by the fact that cell populations form a continuum along the progenitor-glia trajectory and that *in vitro*, mature glia can be induced to de-differentiate and subsequently differentiate into neurons.

Finally, integration of TrajectoryGeometry results with RNA velocity analysis (29) may present an interesting avenue for future exploration. We hypothesise that those cells in the branching region of the default trajectory which are about to branch off will already have changed their velocity. Clustering the velocities of cells in this region might enable inference of their ultimate fate. Further, we propose that genes differentially expressed by cells that have changed their velocity might represent early indicators of cell fate determination.

We have shown how TrajectoryGeometry can detect whether the developmental trajectories leading from bipotential progenitor cells branch or bifurcate and in turn give highly specific information about the genes and functional pathways involved. We are now in an era where there is an explosion in the availability of scRNAseq data and we expect TrajectoryGeometry to facilitate novel insights into cell lineage decisions.

## Supplementary Material

lqae139_Supplemental_File

## Data Availability

TrajectoryGeometry is available as an R Bioconductor package (10.18129/B9.bioc.TrajectoryGeometry). Data describing the development of hepatocytes into hepatoblasts and cholangiocytes (9) was obtained from GEO under the accession GSE90047 and data describing the development of postnatal murine olfactory stem cells into sustentacular cells, neurons and microvillous cells was obtained from GEO under the accession GSE95601. Scripts to reconstruct olfactory trajectories as presented in (10) were obtained from https://github.com/rufletch/p63-HBC-diff. Scripts used to construct hepatoblast trajectories are available at https://github.com/AnnaLaddach/TrajectoryGeometryData and https://zenodo.org/doi/10.5281/zenodo.13837601.
