# Supplementary material for: TrajectoryGeometry suggests cell fate decisions can involve branches rather than bifurcations
Source: NAR Genom Bioinform. 2024 Oct 8;6(4):lqae139. doi: 10.1093/nargab/lqae139 (PMC11459380; doi:10.1093/nargab/lqae139)
Supplement: lqae139_Supplemental_File [file lqae139_supplemental_file.pdf]

Supporting Information

We give an overview of the geometry which enables our analysis. This has been previously described in [1] and in the documentation of [2].

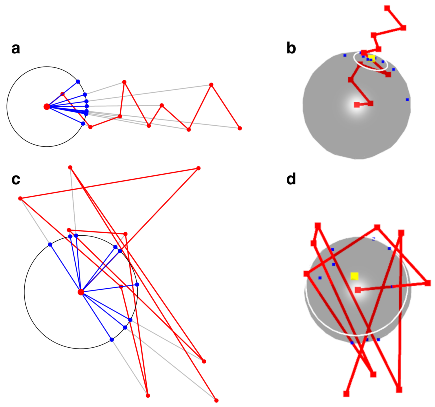

Supplementary Figure 1. Synthetic data. **a)** A path with a well-defined directionality shown in two dimensions. Directions are sighted from its origin producing a well-clustered set of points on the circle. **b)** The same path shown in three dimensions. Here the directions are the blue points on the sphere. Their common center is shown in yellow. The white circle shows their mean distance from this center. **c)** A path without a well-defined direction. Directions to the points of this path are spread out on the circle. **d)** The same path shown in three dimensions. The white circle showing mean distance to the common center is large, reflecting the lack of common directionality.

Directionality in $\mathbb{R}^n$

We would like to analyse whether a given developmental trajectory proceeds in a well-defined direction. This breaks down into two questions: first, does it *have* a well-defined direction and second, does it *proceed* in that direction. We have illustrated the first of these questions in Supplementary Figure 1. In Supplementary Figure 1 **a)** and **b)**, we see a path with a well-defined directionality, in panels **c)** and **d)** we see a path without a well-defined directionality. These two situations are reflected in the clustering or otherwise of the points on the circle and sphere. More generally, in N dimensional space, $\mathbb{R}^N$, a direction is a point on the $N-1$ dimensional sphere $\mathbb{S}^{N-1} = \{x \in \mathbb{R}^N \mid ||x|| = 1\}$.

Our methods are agnostic as to the value of N and to the nature of the N-dimensional space we are working in. In particular, these methods work equally well when working directly with gene expression values or with principal components (PCs) for these values. For expository purposes, we now suppose that we are working with the first 15 principal components of gene expression, so that we are working in $\mathbb{R}^N$, $N = 15$. (In our analyses, we have used $N = 3$ and $N = 10$.) We suppose that we have a pseudotime trajectory as determined by standard pseudotime methods, e.g., by Slingshot [3]. Suppose we now sample 10 cells from a particular trajectory. Call these $\mathbf{c}_1, \ldots, \mathbf{c}_{10}$. Using our first 15 PCs, we then have points $\mathbf{x}_1, \ldots, \mathbf{x}_{10}$ in $\mathbb{R}^{15}$. Sighting from the first point $\mathbf{x}_1$ to each of the successive points $\mathbf{x}_2, \ldots, \mathbf{x}_{10}$ gives us 9 directions in $\mathbb{R}^{15}$, i.e., 9 points on $\mathbb{S}^{14}$. Call these points $\mathbf{p}_1, \ldots, \mathbf{p}_9$. To measure the directionality of the path $\mathbf{x}_1, \ldots, \mathbf{x}_{10}$ (and therefore the directionality of the pseudotime trajectory) we measure how closely $\mathbf{p}_1, \ldots, \mathbf{p}_9$ cluster on $\mathbb{S}^{14}$. That is, we find a center c on $\mathbb{S}^{14}$ that minimizes mean spherical distance to these points. Charmingly, this measure of directionality can be seen as the radius of an $N-2$-sphere on $\mathbb{S}^{N-1}$.

Spherical distance between two points $\mathbf{a}$ and $\mathbf{b}$ of $\mathbb{S}^{14}$ is given by the angle between them, which can be found as arcsine of their dot product. The center c is found by minimizing an objective function which can be either the mean distance or the median distance from c to the points $\mathbf{p}_1, \ldots, \mathbf{p}_9$. Optimization is carried out by applying the R function optim using the chosen objective function (together with a penalty for departing the unit sphere) and a starting point given by taking the (vector) mean of the points $\mathbf{p}_1, \ldots, \mathbf{p}_9$ and normalizing this mean. The resulting mean or median distance from the resulting center c is taken as a measure of how closely the points $\mathbf{p}_1, \ldots, \mathbf{p}_9$ cluster with a smaller distance demonstrating closer clustering.

We have referred here, to our PCA data simply as being in $\mathbb{R}^{15}$, but in fact PCA gives a specific 15-dimensional affine subspace V of $\mathbb{R}^E$, gene expression space. Specifically, it is the unique 15-dimensional subspace with the property that orthogonal projection onto this space minimizes the loss of variation. It is worth noting that while our directionality results are calculated using coordinates given by the individual PCs that form a basis for the space $V \cong \mathbb{R}^{15}$, the results themselves are metric and depend only on V and not on the bases.

We encourage users who are interested in eliminating the sizes of individual cell populations from their analysis to subsample their data set or to perform the analysis in gene expression space.

Testing for directionality

We would like a statistical method for determining when we have discovered statistically significant clustering on $\mathbb{S}^{14}$. Let us take r to be the minimal mean (or median) radius found in the previous paragraph. We can think of r as a function of the original trajectory

$$\mathbf{x}_1, \ldots, \mathbf{x}_{10} \mapsto \mathbf{p}_1, \mathbf{p}_2, \ldots, \mathbf{p}_9 \mapsto r.$$

We refer to this radius as the *clustering radius*.

Each time we sample the pseudotime trajectory, we will get a different value for this radius. In this way, repeated sampling (say 1000 times) produces an empirical distribution of estimates of the directionality of the pseudotime trajectory, $r_1, \ldots, r_{1000}$. We would now like to use this to estimate a p-value for the directionality of this trajectory.

In order to do this, we turn to permutation testing. That is, we produce (say 1000) randomized trajectories $\mathbf{x}^i_1, \ldots, \mathbf{x}^i_{10}$. (We describe the randomization procedure below.) For each of these 1000 randomized trajectories we compute their clustering radius

$$s_i = r(\mathbf{x}^i_1, \ldots, \mathbf{x}^i_{10}).$$

Comparing the values $r_1, \ldots, r_{1000}$ to s $s_1, \ldots, s_{1000}$ using the Wilcoxon signed rank test using the R function wilcox.test gives us a p-value for the clustering on the sphere and therefore, for the directionality of the trajectory we have sampled.

If we detect significant directionality we can proceed to estimate the trajectory's direction in gene expression space. Having sampled the trajectory 1000 times, we find 1000 centers, $c_1, \ldots, c_{1000}$, each the center for one of the sampled

trajectories. We take their common center $\hat{c}$ to give the direction of the overall trajectory. If we are working with the first 15 PCs, this will be a unit vector in $\mathbb{R}^{15}$. This can then be translated back into gene expression space where each coordinate represents a gene. The most positive and most negative coordinates in this vector reveal the genes which are most strongly up- and down-regulated along this trajectory. In this way, directionality testing can be used to reveal biologically significant information.

Notice that trajectories are typically paths between cell clusters. Thus, while the use of PCA will filter some of the gene expression information, in the case where we use a sufficient number of PCs to capture a substantial portion of the overall variation, we would expect the most prominent genes in the overall trajectory to survive dimension reduction, as is observed in the analyses presented here.

There are multiple ways of producing a randomized trajectory. The most conservative of these is via matrix permutation. We can consider the path $\mathbf{x}_1, \ldots, \mathbf{x}_{10}$ as a matrix X where each of these points is a row of X. Thus X has dimension 10×15. We can then independently randomly permute the entries in each column of X thus independently permuting the time order for each PC (or gene in the case where we are working directly with gene expression). We describe this as conservative in that it conserves the distribution in each coordinate. A less conservative method is to randomly permute the entries of X without respect to columns. Further details and other methods of producing randomized paths are described in [1] and in the vignette accompanying TrajectoryGeometry [2] on Bioconductor.

Hepatoblasts, hepatocytes and cholangiocytes: directionality in full gene expression space

Here we report directionality analysis of gene trajectories in the data from [4] as above, using higher dimensional gene expression data, namely, the 17583 that remain after filtering out all genes expressed in 10 or fewer cells. These results are shown in Supplementary Figure 2. Supplementary Figure 2a shows mean spherical distance on the 17582-sphere for the hepatoblast to hepatocyte trajectory sampled 1000 times compared to 1000 randomised trajectories. We see that the directionality of this trajectory in this higher dimensional space is highly statistically significant. Supplementary Figure 2b shows the results of a similar analysis for the hepatoblast to cholangiocyte trajectory. These results parallel those of Figure 2d. Supplementary Figure 2c shows the comparison between the directionality of the hepatoblast to hepatocyte trajectory and that of the hepatoblast to cholangiocyte trajectory as measured by their clustering radii on the 17582-sphere. These results parallel those shown in Figure 2e, and show that the hepatocyte trajectory maintains a significantly more consistent directionality than the cholangiocyte trajectory. Gene scores for the association of genes with the directionality of the trajectory in PCA space are highly correlated with those in gene expression space for the 1559 genes that are common between the two analyses (Spearman's $\rho = 0.951$ cholangiocyte trajectory; Spearman's $\rho = 0.946$ hepatocyte trajectory). Indeed, the top 10 up- and down-regulated genes for each trajectory, from the analysis in 10-dimensional PCA space (Figure 2c) show similar trends to those calculated in 17582-dimensional gene expression space (Figure 2d-e). Taken together, these results suggest that this sort of directionality analysis is robust with respect to dimensionality and support the use of principal component data to estimate the directionality of pseudotime trajectories.

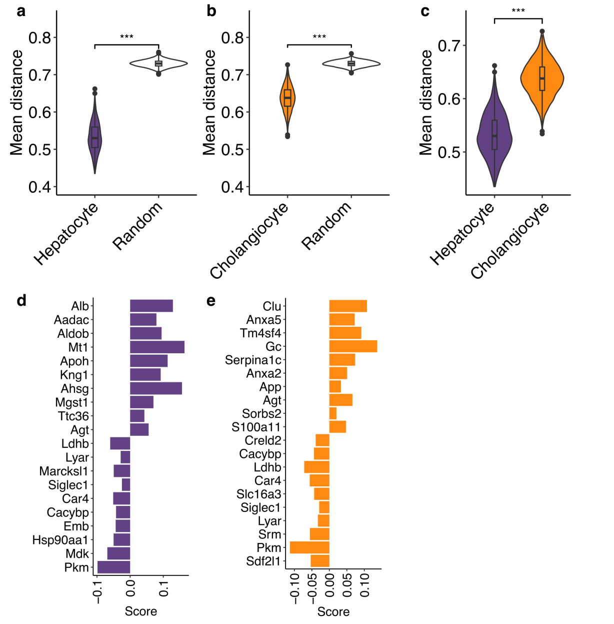

Supplementary Figure 2. Directionality of trajectories using the full gene expression space $\mathbb{R}^{17583}$. **a)** Comparison of directionality in $\mathbb{S}^{17582}$ for hepatoblast to hepatocyte trajectory, 1000 samples. **b)** Comparison of directionality in $\mathbb{S}^{17582}$ for hepatoblast to cholangiocyte trajectory, 1000 samples. **c)** Comparison of directionality in $\mathbb{S}^{17582}$ for the hepatoblast to hepatocyte and hepatoblast to cholangiocyte trajectories. **d)** Bar plot showing top 10 up-and down-regulated genes for the hepatoblast to hepatocyte trajectory in $\mathbb{S}^{17582}$. **e)** Bar plot showing top 10 up- and down-regulated genes for the hepatoblast to cholangiocyte trajectory in $\mathbb{S}^{17582}$.

Nested cell fate decisions

Data from [3] offer an opportunity to investigate nested cell fate decisions. Supplementary Figure 3a), a plot in PCA space, shows postnatal murine olfactory stem cells (also called horizontal basal cells (HBCs)) giving rise to sustentacular cells, neurons and microvillous cells (MVCs). Visual inspection of this plot suggests that the HBC to Sustentacular trajectory is a default trajectory, consistent with the fact that the latter are produced via direct fate conversion from HBCs. In contrast both neurogenic and MVC trajectories appear to branch off from the sustentacular trajectory at the first decision point (DP1), before diverging from one another at a second decision point (DP2) corresponding to the globose basal cell state (GBCs).

DP1 is a branch point

Focussing initially on the neuronal/sustentacular fate decision, TrajectoryGeometry analysis reveals that although both trajectories show significant directionality in comparison to randomised trajectories (Supplementary Figure 3d), the sustentacular trajectory displays a more consistent directionality relative to the neuronal (and microvillous) trajectory (Supplementary Figure 3b,d). Genes with a positive score for the sustentacular trajectory include sustentacular markers (e.g. Cyp2g1 [3]) whereas those with a negative

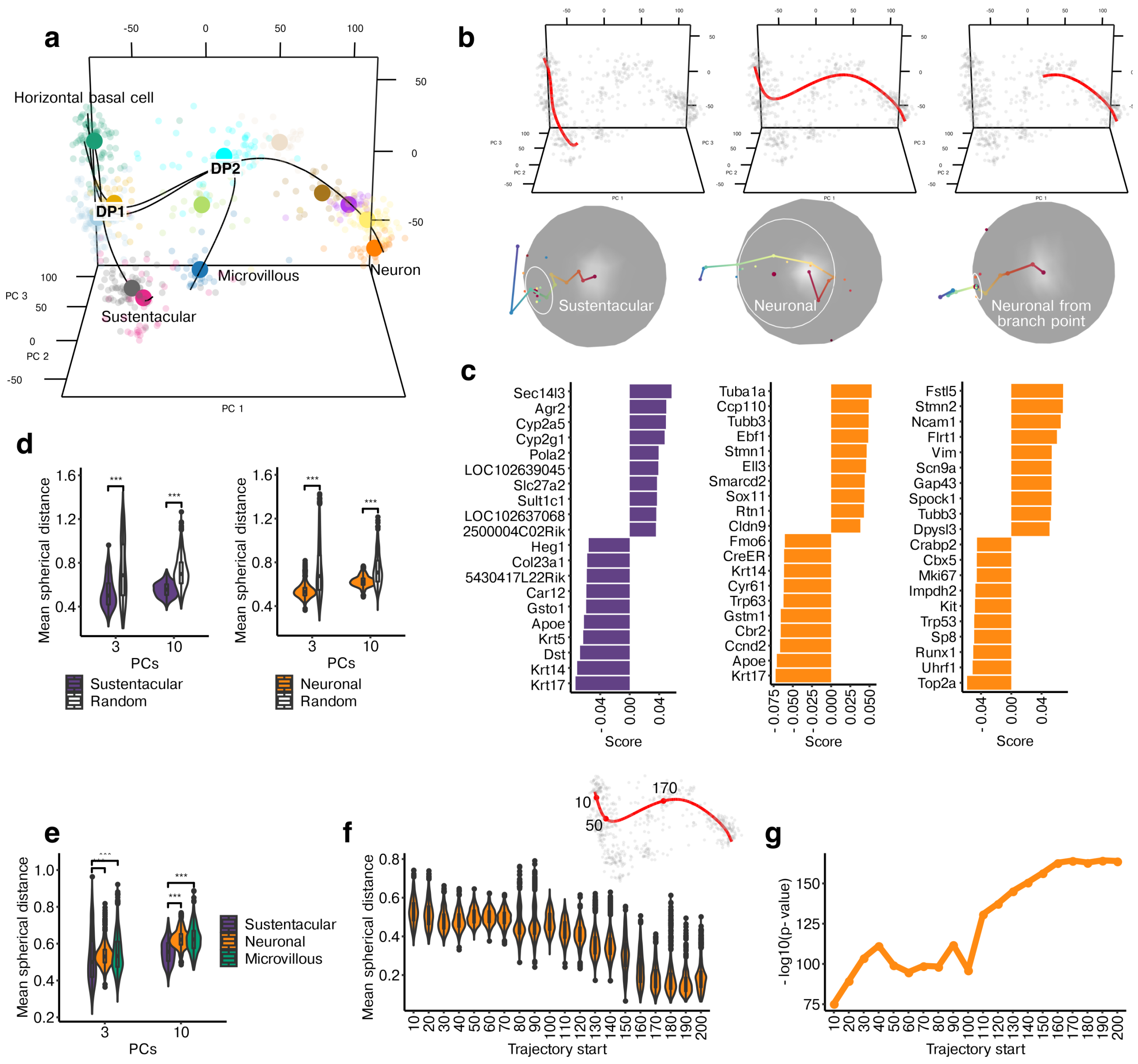

Supplementary Figure 3. a) PCA plot of scRNAseq data for adult murine olfactory cells. Pseudotime trajectories inferred using Slingshot as in [3] are shown on the plot. Cells are coloured by cluster as inferred in [3]. **b)** 3-dimensional sampled pathways for HBC to sustentacular, HBC to neuron, and DP2 to neuron trajectories together with their projections on the 2-sphere. White circles denote mean distance from center (red dot). **c)** Bar plots showing top 10 up-and down-regulated genes for each trajectory as in b). **d)** Violin plots indicating the mean spherical distance (radii of the white circles in b) for paths sampled from the sustentacular and neuronal trajectories (purple and orange, respectively) relative to random trajectories (white). Statistics calculated using 1000 random paths from each trajectory and the first 3 and the first 10 PCs respectively. **e)** Violin plots indicating the mean spherical distance of the sustentacular (purple), neuronal (orange) and microvillous (green) trajectories using 1000 random paths from each trajectory and the first 3 and the first 10 PCs respectively. **f)** Violin plots indicating the mean spherical distance for the neuronal trajectory (first 3 PCs) starting from successively later points in pseudotime, as DP1 and DP2 are approached (values 50 and 170 on the neuronal trajectory shown in the top right inset). **g)** Line graph indicating the −log10(p-value) for the significance of directionality for the neuronal trajectory (first 3 PCs) relative to random trajectories, starting from successively later points in pseudotime.

score for the directionality of the sustentacular and neuronal trajectories include HBC stem cell markers (Krt14, Krt5, Trp63 [3] (Supplementary Figure 3c)). Although the top scored genes for the overall neurogenic trajectory HBC-Neurons) include neurogenic markers Sox11 and Tubb3 [5], GO term overrepresentation analysis [6] reveals that the top 5 % of genes with positive scores for this trajectory are highly enriched for cell cycle markers (Supplementary Figure 4a), suggesting that this directionality does not lead to the mature neuronal

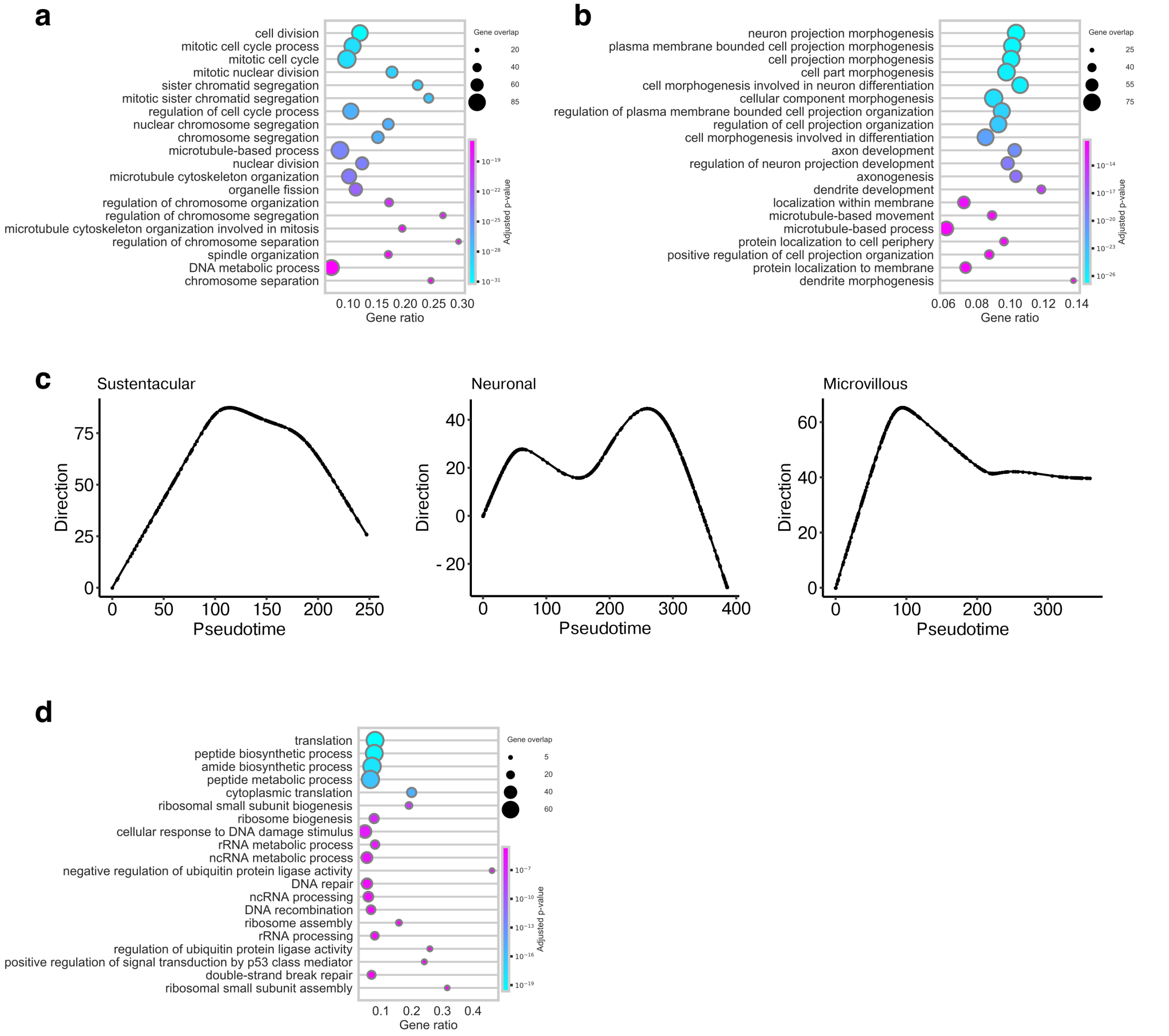

Supplementary Figure 4. a) Dot plot showing GO terms overrepresented among the top 5 % of genes associated with the HBC-neuron directionality. Dot size indicates the overlap for each term, and gene ratio indicates the fraction of genes in each term. **b)** As in a) for genes associated with the DP2-neuron directionality. **c)** Line graphs showing the progress of smoothed trajectories projected onto PC4. **d)** As in a) for the top 5% of genes associated with PC4.

phenotype and may be strongly influenced by the proliferative GBC population at DP2.

In spite of the relatively more consistent directionality of the sustentacular trajectory, projection onto principle components reveals that it makes a U-turn in PC4 (Supplementary Figure 4c). Interestingly GO term overrepresentation analysis of the top 5% of genes [6] shows this PC is highly associated with ribosomal genes and protein synthesis (Supplementary Figure 4d), suggesting transient upregulation of these genes is required for the synthesis of proteins required by the emergent cell type. Indeed, a similar pattern is also seen for the later neuronal (and microvillous) trajectories (Supplementary Figure 4d), suggesting that this is a common characteristic coincident with differentiation.

Intriguingly, the trajectory from the first decision point to neurons is not straight (see below). Furthermore, Supplementary Figure 3g shows that the trajectory from progenitors to neurons becomes more directional after passing the first decision point (at value 50 on the neuronal trajectory) and again after passing the second decision point (at value 170 on the neuronal trajectory) (Supplementary Figure 3b,f,g). Interestingly the top 5 % of genes associated with the segment of the neuronal trajectory from the second decision point onwards (DP2-Neurons) (Supplementary Figure 3c) are highly enriched [6] in GO terms associated with the development

of a mature neuronal phenotype, such as axonogenesis and neuron projection development (Supplementary Figure 4b). Those negatively associated with the directionality of this post-DP2 trajectory segment include cell cycle markers (e.g. Top2a, Mki67), indicating progress in this direction involves departure from the cycling GBC state at DP2.

DP2 exhibits branching behaviour

We now shift our focus to DP2, the neuronal/microvillous decision (Supplementary Figure 5a).

Specifically we study the microvillous cell (MVC) and neuronal trajectories from DP1 onwards, and subdivide them at DP2 (Supplementary Figure 3a). Interestingly neither DP1-Neurons nor DP1-MVCs is straight and the directionality of both trajectories becomes more significant after DP2 (Supplementary Figure 5a,b,d,e). Comparing the directions of their initial and final segments (using the first 10 PCs), DP1-Neurons turns approximately 124 degrees and DP1-MVCs makes a turn of 126 degrees (Supplementary Figure 3a depicts the first 3 PCs.) Therefore on initial inspection, DP2 is a bifurcation with each trajectory initiating a new transcriptomic programme.

Notice that by turning more than 90 degrees each of these has partially reversed direction, indicating the partial retraction of a transcriptomic programme. Supplementary Figure 5c and g show the progression of the pseudotime trajectories for DP1-MVCs and DP1-Neurons in the direction defined by DP1-DP2. Here it can be seen that progress is reversed after the decision point suggesting DP2 is a transient state.

To identify the transiently upregulated genes, we considered the top 5% up- and down-regulated genes in the directions for DP1-DP2, DP2-MVCs and DP2-Neurons and looked at the intersection of the genes that were up-regulated in the first leg with those that were down in the second leg. GO term overrepresentation analysis [6] showed that such genes transiently upregulated for both the microvillous and the neuronal trajectories were highly enriched for cell-cycle associated terms (Supplementary Figure 5g, Supplementary Supplementary Figure 6a), consistent with the proliferative nature of GBCs. This suggested that change in direction observed for both neuronal and microvillous trajectories was dominated by reentry into and departure from the cell cycle.

To test the hypothesis that branching behaviour was being obscured by cell-cyle effects, we reanalysed data from DP1 onwards, omitting cell-cycle associated PC2 (Supplementary Supplementary Figure 6b, c). Interestingly, this showed the neuronal trajectory to have significantly more consistent directionality than the MVC trajectory (Supplementary Figure 5h). Therefore if cell-cycle effects are not considered, DP1 appears to be a branch point with the microvillous trajectory branching off from the neuronal trajectory. Put differently, the geometry observed at DP2 results from the transient overlay of cell-cycle on branching behaviour. Furthermore, the top genes associated with the DP1-neuron directionality are enriched in terms that indicate acquisition of a mature neuronal phenotype (e.g. axonogenesis) if PC2 is omitted (Supplementary Supplementary Figure 6c, d).

Taken together, these results support the hierarchical branching of trajectories, with the neurogenic and MVC trajectories first branching off a default sustentacular trajectory. Although both MVC and neuronal trajectories then enter the cycling GBC state, the neuronal trajectory appears to be a default upon exit of the cell cycle, whereas the microvillous trajectory branches off, suggesting it may require the input of more extrinsic signals. As microvillous cells are comparatively rare it is parsimonious that these are not produced by default. Importantly, by considering the contribution of individual PCs to directionality we were able to gain insight into the dynamics of cell-type specific transcriptional programmes, and generic transcriptional programmes (translation, cell-cycle).

A negative control

In order to provide a negative control for the detection of branching behaviour by our software, we created synthetic data which exhibits a symmetric bifurcation. Supplementary Figure 7a) shows the first 2 PCs for this data with the “cells” coloured by Seurat cluster and the two trajectory curves superimposed. Both of these trajectories show highly statistically significant directionality when compared to randomized trajectories, both in dimension 3 and in dimension 10 as shown in Supplementary Figure 7 b) and c). However, they are missing telltale signs of branching behaviour. The first of these signs would be that one of the trajectories is more directional than the other. However, their degree of statistical significance is comparable. The underlying measure of their directionality is the degree of spherical clustering of their projections. Again, we see no significant difference in these values either in dimension 3 or dimension 10 (Supplementary Figure 7d), compare Figure 2d.) Finally, if there were branching behaviour on one of these trajectories, we would expect that when starting at later pseudotimes, we would see an increase in significance up to the point when we start somewhere near the decision point, as we see in Figure 2g. However, in Supplementary Figure 7e, we see a loss of significance as we approach normalized pseudotime 40, slightly before the decision point. We attribute this to the comparison between the overall noise level in the data and the remaining length of the chosen path. Significance increases after the decision point, but equally for both curves, again suggesting the absence of a default trajectory along one and branching behaviour on the other.

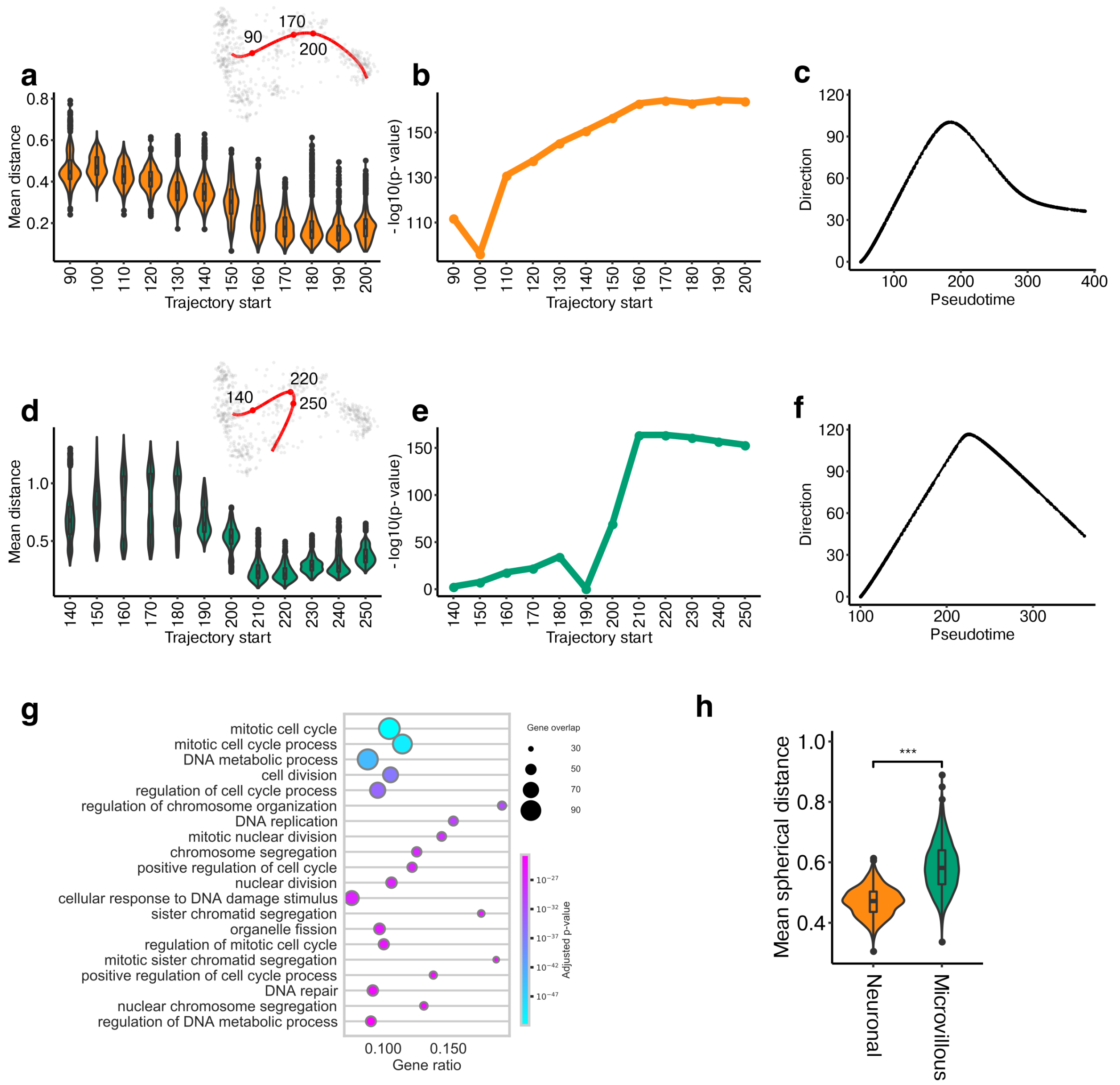

Supplementary Figure 5. **a)** Violin plots indicating the mean spherical distance for the neuronal trajectory (first 3 PCs) starting from successively later points in pseudotime, as DP2 is approached (value 170 on the neuronal trajectory shown in the top right inset). **b)** Line graph indicating the –log10(p-value) for the significance of directionality for the neuronal trajectory (first 3 PCs) relative to random trajectories, starting from successively later points in pseudotime. **c)** Line graph showing progress of the smoothed neuronal trajectory (available in 5 PCs) projected onto the DP1-DP2 directionality. **d)** Violin plots indicating the mean spherical distance for the microvillous trajectory (first 3 PCs) starting from successively later points in pseudotime, as DP2 is approached (value 220 on the microvillous trajectory shown in the top right inset). **e)** Line graph indicating the –log10(p-value) for the significance of directionality for the microvillous trajectory (first 3 PCs) relative to random trajectories, starting from successively later points in pseudotime. **f)** Line graph showing progress of the smoothed microvillous trajectory (available in 5 PCs) projected onto the DP1-DP2 directionality. **g)** Dot plot showing GO terms overrepresented among the top 5 % of transiently upregulated genes at DP2 for the microvillous trajectory. Dot size indicates the overlap for each term, and gene ratio indicates the fraction of genes in each term. **h)** Violin plots indicating the mean spherical distance of the neuronal (orange) and microvillous (green) trajectories from DP1 onwards using 1000 random paths from each trajectory and PCs 1, 3-10 (omitting PC2).

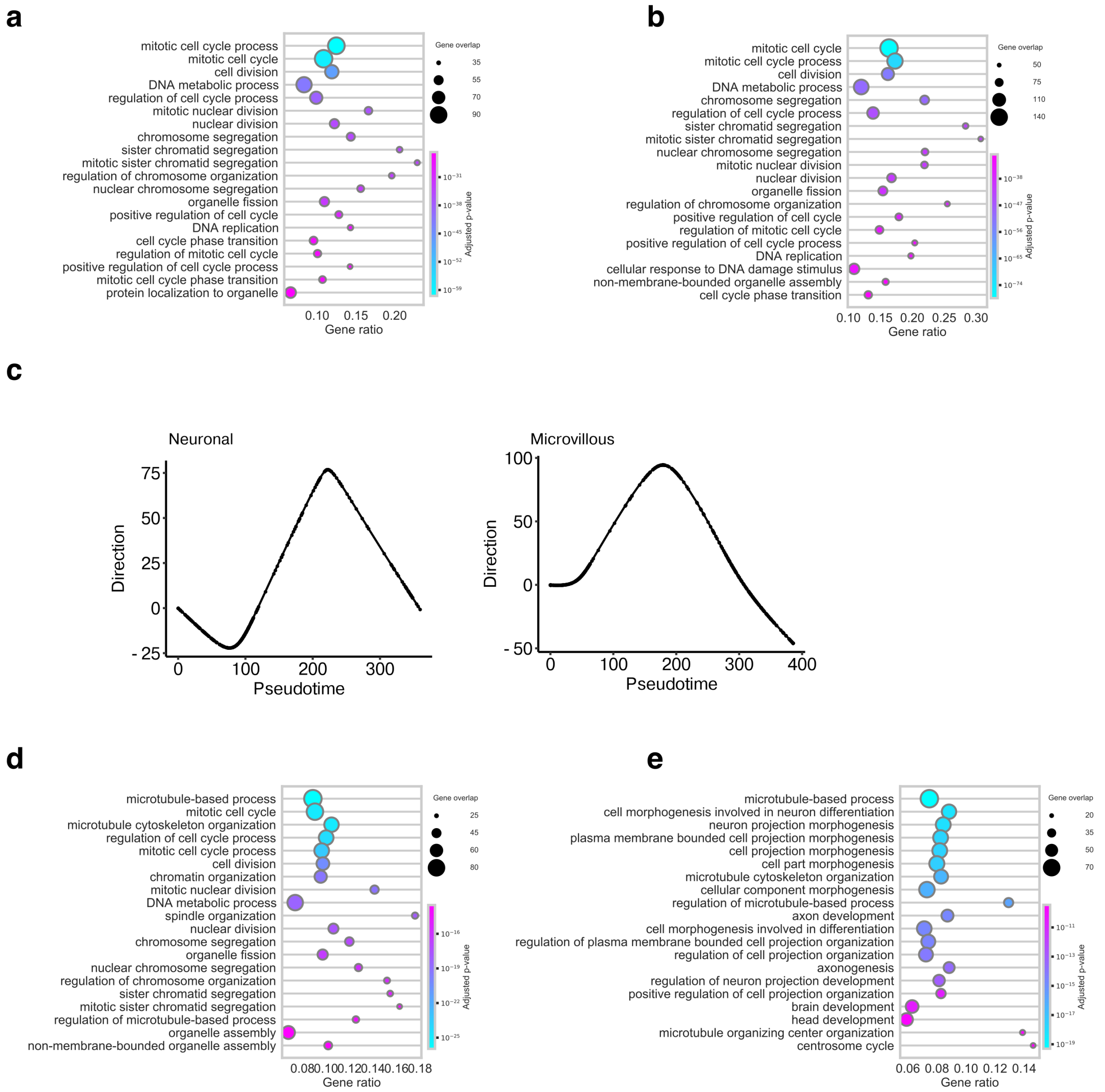

Supplementary Figure 6. a) Dot plot showing GO terms overrepresented among transiently upregulated genes at DP2 for the neuronal trajectory (the intersection of the top 5% up-regulated genes in the DP1-DP2 direction with the top 5% of downregulated genes in the DP2-Neurons direction). Dot size indicates the overlap for each term, and gene ratio indicates the fraction of genes in each term. **b)** As in a) for the top 5% of genes associated with PC2. **c)** Line graphs showing the progress of smoothed trajectories projected onto PC2. **d)** As in a) for the top 5% of genes associated with the DP1-neuron directionality. **e)** As in a) for the top 5% of genes associated with the DP1-neuron directionality omitting PC2.

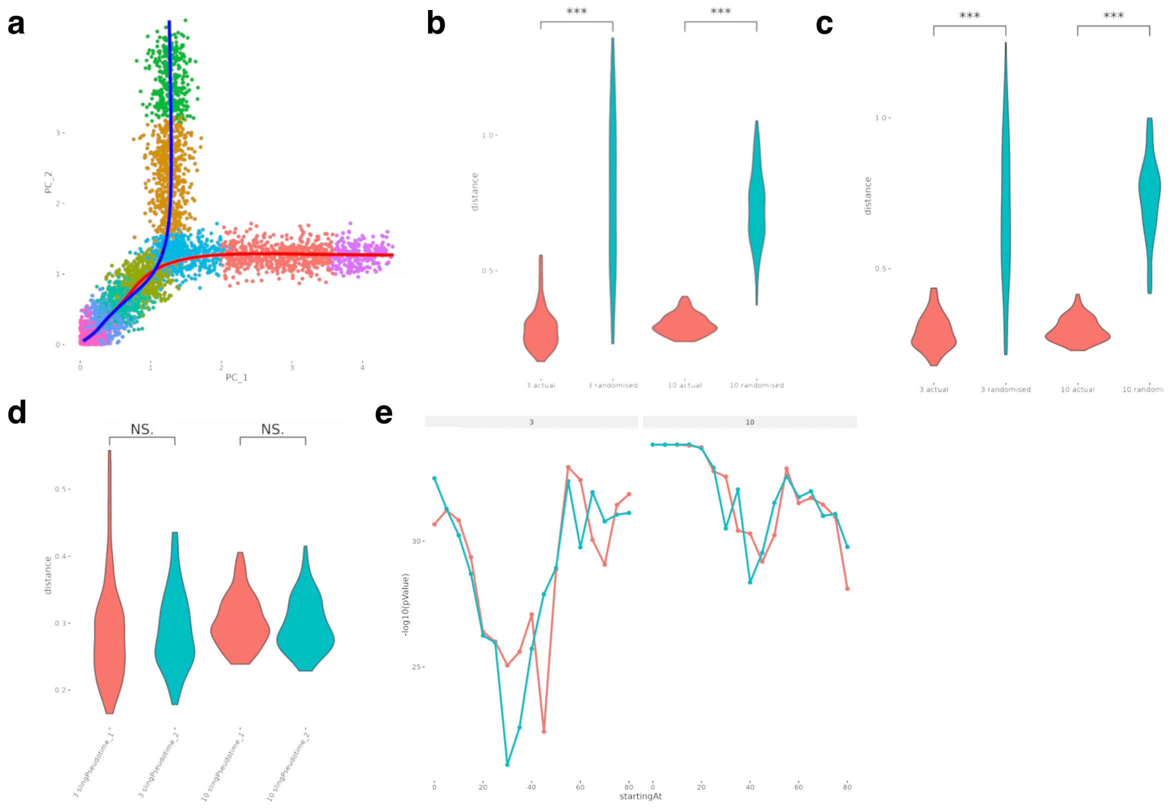

Supplementary Figure 7. a) PCA plot of synthetic data with symmetric bifurcation. Points are colored by Seurat cluster. Trajectory curves 1 and 2 are red and blue respectively. **b)** and **c)** Both trajectories are significantly directional when compared to randomized trajectories. We make these comparisons using the first 3 PCs and the first 10 PCs. **d)** The directionality of the two trajectories as measured by spherical clustering in dimensions 3 and 10 show no significant difference. **e)** We show p-value for the trajectories in dimensions 3 and 10 when sampling starting at successively later points in normalized pseudo-time.

References

1. Anna Laddach, Song Hui Chng, Reena Lasrado, Fränze Progatzky, Michael Shapiro, Alek Erickson, Marisol Sampedro Castaneda, Artem V. Artemov, Ana Carina Bon-Frauches, Eleni-Maria Amaniti, Jens Kleinjung, Stefan Boeing, Sila Ultanir, Igor Adameyko, and Vassilis Pachnis. A branching model of lineage differentiation underpinning the neurogenic potential of enteric glia. *Nature Communications*, 14(1):5904, Sep 2023.
2. Anna Laddach and Michael Shapiro. TrajectoryGeometry. *www.bioconductor.org*, 2021. http://www.bioconductor.org/packages/release/bioc/html/TrajectoryGeometry.html. Last accessed 2024-08-15.
3. Russell B Fletcher, Diya Das, Levi Gadye, Kelly N Street, Ariane Baudhuin, Allon Wagner, Michael B Cole, Quetzal Flores, Yoon Gi Choi, Nir Yosef, Elizabeth Purdom, Sandrine Dudoit, Davide Risso, and John Ngai. Deconstructing olfactory stem cell trajectories at single-cell resolution. *Cell stem cell*, 20(6):817–830.e8, Jun 2017.
4. L Yang, WH Wang, WL Qiu, Z Guo, E Bi, and CR Xu. A single-cell transcriptomic analysis reveals precise pathways and regulatory mechanisms underlying hepatoblast differentiation. *Hepatology*, 66(5)):1387–1401, 2017.
5. Li Sha, Rob Kitchen, David Porteous, Douglas Blackwood, Walter Muir, and Benjamin Pickard. Sox11 target genes: implications for neurogenesis and neuropsychiatric illness. *Acta neuropsychiatrica*, 24(1):16–25, Feb 2012.
6. Uku Raudvere, Liis Kolberg, Ivan Kuzmin, Tambet Arak, Priit Adler, Hedi Peterson, and Jaak Vilo. g:profiler: a web server for functional enrichment analysis and conversions of gene lists (2019 update). *Nucleic acids research*, 47(W1):W191–W198, Jul 2019.
